# Reliability of Multiparametric Magnetic Resonance Imaging in Patients with a Previous Negative Biopsy: Comparison with Biopsy-Naïve Patients in the Detection of Clinically Significant Prostate Cancer

**DOI:** 10.3390/diagnostics13111939

**Published:** 2023-06-01

**Authors:** Biagio Barone, Luigi Napolitano, Francesco Paolo Calace, Dario Del Biondo, Giorgio Napodano, Marco Grillo, Pasquale Reccia, Luigi De Luca, Domenico Prezioso, Matteo Muto, Felice Crocetto, Matteo Ferro

**Affiliations:** 1 Department of Neurosciences, Reproductive Sciences and Odontostomatology, University of Naples “Federico II”, 80131 Naples, Italy; 2 Unit of Urology, Hospital “Ospedale del Mare”, ASL Napoli 1 Centro, 80147 Naples, Italy; 3 Department of Medical-Surgical Biotechnologies and Translational Medicine, University of Rome Tor Vergata, 00133 Rome, Italy; 4 Department of Onco-Hematological Diseases, AORN “San Giuseppe Moscati”, 83100 Avellino, Italy; 5 Division of Urology, European Institute of Oncology IRCSS, 20141 Milan, Italy

**Keywords:** prostate cancer, prostate biopsy, magnetic resonance imaging

## Abstract

**Background**: Multiparametric magnetic resonance is an established imaging utilized in the diagnostic pathway of prostate cancer. The aim of this study is to evaluate the accuracy and reliability of multiparametric magnetic resonance imaging (mpMRI) in the detection of clinically significant prostate cancer, defined as Gleason Score ≥ 4 + 3 or a maximum cancer core length 6 mm or longer, in patients with a previous negative biopsy. **Methods**: The study was conducted as a retrospective observational study at the University of Naples “Federico II”, Italy. Overall, 389 patients who underwent systematic and target prostate biopsy between January 2019 and July 2020 were involved and were divided into two groups: Group A, which included biopsy-naïve patients; Group B, which included re-biopsy patients. All mpMRI images were obtained using three Tesla instruments and were interpreted according to PIRADS (Prostate Imaging Reporting and Data System) version 2.0. **Results**: 327 patients were biopsy-naïve, while 62 belonged to the re-biopsy group. Both groups were comparable in terms of age, total PSA (prostate-specific antigen), and number of cores obtained at the biopsy. 2.2%, 8.8%, 36.1%, and 83.4% of, respectively, PIRADS 2, 3, 4, and 5 biopsy-naïve patients reported a clinically significant prostate cancer compared to 0%, 14.3%, 39%, and 66.6% of re-biopsy patients (*p* < 0.0001–*p* = 0.040). No difference was reported in terms of post-biopsy complications. **Conclusions**: mpMRI confirms its role as a reliable diagnostic tool prior to performing prostate biopsy in patients who underwent a previous negative biopsy, reporting a comparable detection rate of clinically significant prostate cancer.

## 1. Introduction

Prostate cancer (PCa) represents the second leading cause of death and the most frequent cancer in men worldwide, with over 268,490 new estimated cases diagnosed in 2021 [1]. Despite the incidence and mortality of this cancer being strictly related to age, with an increased incidence in elderly men over 65 years, a higher risk for African-American men and for patients with a positive family history of prostate and breast cancer has been similarly reported [2,3]. PCa could display variable and heterogeneous clinical behaviors, being mostly asymptomatic at the early stage [4]. Considering the impact of PCa in the male population, the role of screening and early diagnosis is pivotal in the management of PCa and balancing. However, there is a risk of overdiagnosis and overtreatment of indolent disease [5,6]. The traditional diagnostic pathway of PCa starts from an elevated serum prostate-specific-antigen (PSA) level and/or an abnormal digital–rectal examination (DRE), followed by a transrectal ultrasound examination (TRUS), in the absence of a dedicated and accurate imaging modality [7,8]. Nevertheless, only prostate biopsy (PB) could confirm the suspicion of PCa, even if negative histopathological findings or dubious lesions are frequently encountered in clinical practice, limiting the PCa detection rate at 40–45% [9,10]. The management of patients with a prior negative biopsy represents a clinical challenge, especially in those cases where PCa clinical suspicion persists. While a new biopsy could be performed in this setting, potential side effects, as well as the possibility of missing significant cancers, are present, and a careful balancing between cost, risk, and cancer detection has to be made [11,12]. In the last ten years, the multiparametric prostate magnetic resonance imaging (mpMRI) has been recognized as a reliable and useful imaging technique for PCa detection and localization, providing information on prostate volume, tissue morphology, vascularity, and characteristics, achieving an overall detection rate of 72–75% [13,14,15,16]. As stated by the European Association of Urology (EAU) guidelines, mpMRI before prostate biopsy could improve the detection rate of the procedure and, contextually, reduce the number of unnecessaries biopsies [17]. As result, mpMRI has been increasingly used to identify patients, which could require a repeated biopsy and identify specific anatomic targets. The aim of our study is therefore to evaluate the diagnostic accuracy of mpMRI in the detection of clinically significant and not PCa in patients with a previous negative biopsy compared to naïve patients.

## 2. Materials and Methods

The study was conducted between January 2019 and July 2020 as a retrospective observational study at the University “Federico II” of Naples, Italy, in according with the guidelines of World Medical Association Declaration of Helsinki. Written informed consent was provided by all patients involved in the study. No ethical committee was required due to the retrospective nature of the study and the absence of procedures not included in the normal diagnostic and therapeutic algorithm of the disease. For each patient, we collected biometric information and previous biopsy data. Exclusion criteria were PCa in active surveillance and mpMRI older than 3 months. A total of 389 patients were divided into two distinct groups: group A, which represented naïve patients; Group B, which represented patients with a previous negative biopsy (up to 2 years).

### 2.1. mpMRI Protocol

Patients underwent mpMRI due to clinical suspicion of PCa, i.e., PSA > 4 ng/mL or positive digital rectal exam. All mpMRIs were performed with a 3 Tesla scanner, acquiring axial and sagittal T1, T2 weighted imaging, and axial diffusion-weighted imaging (DWI). The results were reported according to Prostate Imaging—Reporting and Data System 2015, Version 2 (PIRADS). All mpMRIs were interpreted and scored independently by two different experienced genitourinary radiologists.

### 2.2. Biopsy Protocol

Biopsies were performed following a standardized protocol under TRUS guidance. Indications for prostate biopsy were based on PIRADS ≥ 3 and, for PIRADS 2, in the case of persisting clinical suspicion for PCa (PSA > 4 ng/mL and/or positive DRE). All patients were prescribed a pre-biopsy dose of antibiotics (Ceftriaxone 2 g i.m), while a periprostatic lidocaine infiltration nerve blockade was performed before the procedure. A systematic biopsy protocol, including at least 12 cores with the addition of an eventual 3 target that was cognitively obtained, was performed. As no general agreement has been reported on the definition of clinically significant prostate cancer (csPCa), we defined CSPCa as a Gleason score (GS) ≥ 4 + 3 and/or a maximum cancer core length ≥ 6 mm, in accordance with the prostate MRI imaging study (PROMIS) [18].

### 2.3. Pathologic Analysis

Histopathologic examination was carried out by two experienced genitourinary pathologists. Specimens were processed according to the routinary formalin solution fixation and reports were delivered as GS and ISUP grade. When more cores detected PCa, the highest GS was reported.

### 2.4. Statistical Analysis

Descriptive statistics included mean and standard deviation for continuous variables, while frequencies and percentages were obtained for categorical variables. Categorical variables were evaluated using Pearson’s chi-squared test in order to obtain detection rates, while the Student’s *t*-test was performed for continuous variables among the two groups. Statistical analysis was performed using International Business Machines Corporation Statistical Product and Service Solutions (IBM SPSS) software for Windows (version 25.0., IBM Corp, Armonk, NY, USA). Statistical significance was considered as *p* < 0.05.

## 3. Results

In total, 389 patients were involved in the study, with 327 belonging to the biopsy-naïve group (Group A) and 62 to the re-biopsy group (Group B). Descriptive characteristics of total patients involved are reported in Table 1. Both groups were comparable in terms of age, total PSA, and number of cores obtained at the biopsy. Conversely, a positive DRE was found significantly more frequently in Group A compared to Group B, with 79.6% of positive findings versus 20.4% (*p* = 0.047). The PIRADS score, the side of the lesion at mpMRI, the Gleason score, and the side of the lesion at the subsequent biopsy were comparable among the two groups. Finally, complications among the two groups were comparable, with about 20% of patients in both groups reporting a fever that required antibiotic therapy after biopsy (Table 2).

**Figure 1:**
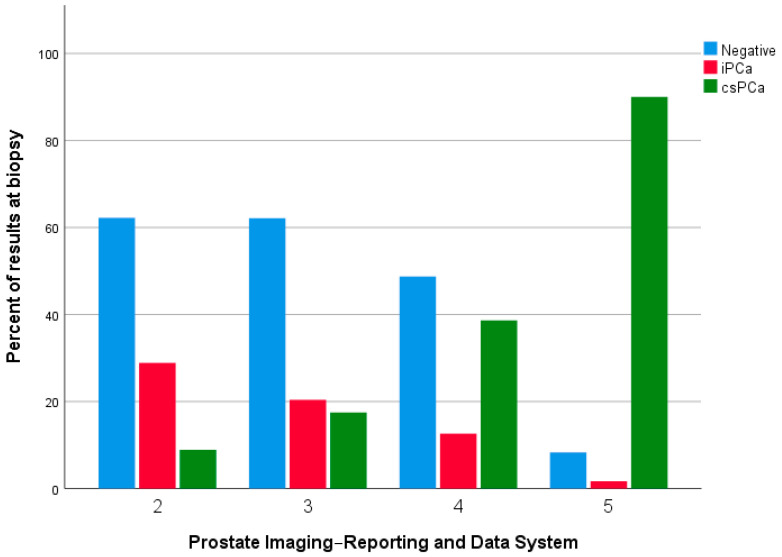
Distribution of results at the biopsy compared to PIRADS score in biopsy-naïve patients. iPCa: Clinically insignificant prostate cancer; csPCa: Clinically significant prostate cancer.

**Figure 2:**
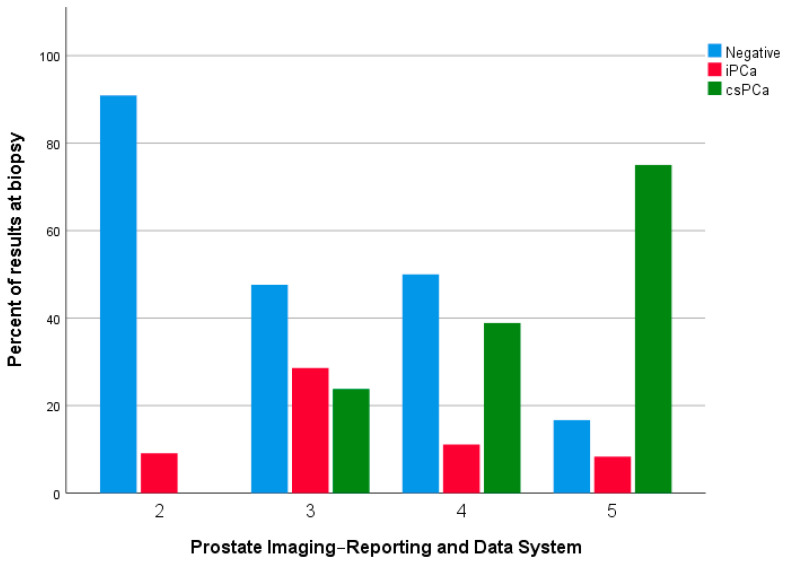
Distribution of results at the biopsy compared to PIRADS score in re-biopsy patients. iPCa: Clinically insignificant prostate cancer; csPCa: Clinically significant prostate cancer.

Table 3 and Table 4 report the data regarding the concordance between PIRADS score at mpMRI and histopathological result at the biopsy and the corresponding ISUP grade. As showed in both tables, 62.2% of PIRADS 2 biopsy-naïve patients reported a negative result, compared to the 90.9% of re-biopsy patients (ISUP grade 0). Conversely, 35.6% of patients in the first group and 9.1% of patients in the second group reported a clinically insignificant PCa (GS 3 + 3 and GS 3 + 4 i.e., ISUP grade 1 and 2), while 2.2% of patients in the first group reported a csPCa (GS 4 + 5 i.e., ISUP grade 5). No patients in the second group reported a csPCa. Analogous results were obtained for PIRADS 3, with 62.1% of group A patients and 47.6% of group B patients reporting a negative result at the biopsy compared to 29% and 38.1% of patients, respectively, who reported a clinically insignificant PCa (PIN, GS 3 + 3 and GS 3 + 4 i.e., ISUP grade 1 and 2). Overall, 8.8% of patients in Group A and 14.3% of patients in group B reported a csPCa (GS 4 + 3, GS 4 + 4, GS 4 + 5, i.e., ISUP grade 3, 4, and 5). For PIRADS 4, 48.7% and 50% of patients in group A and group B, respectively, reported a negative result at the biopsy, compared to 15.1% and 11.2% of patients who reported a clinically insignificant PCa (PIN, GS 3 + 3, and GS 3 + 4, i.e., ISUP grade 1 and 2); 36.1% of Group A patients and 39% of Group B patients reported a csPCa (GS 4 + 3, GS 4 + 4, GS 4 + 5, GS 5 + 4, and GS 5 + 5, i.e., ISUP grade 3, 4, and 5). Finally, regarding PIRADS 5, 8.3% of patients in group A and 16.7% of patients in group B reported a negative result at the biopsy compared to 8.4% and 16.6% of Group A and B patients who reported, respectively, a clinically insignificant PCa (GS 3 + 3 and GS 3 + 4, i.e., ISUP grade 1 and 2). Conversely, 83.4% and 66.6% of Group A and B patients reported a csPCa (GS 4 + 4, GS 4 + 5, GS 5 + 4, GS 5 + 5, i.e., ISUP grade 3, 4, and 5) (*p* < 0.0001 and *p* = 0.040; *p* < 0.0001 and *p* = 0.018) (Figure 1 and Figure 2).

## 4. Discussion

Persistent or increasing PSA levels in patients with previous negative biopsy represent a challenging clinical dilemma. Conventionally, the only way to assess the potential presence of PCa would be a repetition of a systematic TRUS-guided biopsy. Nevertheless, the probability to detect PCa in men with a previous negative biopsy, via a new prostate biopsy, decreases with the increasing number of previous negative biopsies, from 18% to 7% for over two biopsies [19,20]. In addition, although prostate biopsy is generally considered a safe procedure with a relatively low rate of complication, it is not free from risks, and potential infections during and after the prostate biopsy could increase the morbidity of the procedure [21]. As a result, it is crucial to evaluate an alternative, or at least an intermediate imaging method, for patients who underwent a previous negative biopsy but report a persisting clinical suspicion for PCa. The role of mpMRI in the diagnostic pathway of prostate cancer is well-established in biopsy-naïve patients, permitting the revolutionization of the detection of csPCa. In addition, technical development and accumulated data on mpMRI in prostate cancer have permitted us to utilize this imaging technique before re-biopsy in order to potentially solve the limitations of repetitive systematic biopsy. Consistent with these premises, in our study, we evaluated the reliability and the efficacy of mpMRI in detecting PCa and, particularly, csPCa, in a group of patients with a previous negative prostate biopsy compared to biopsy-naïve patients. The rationale of our study resides in a recent AUA consensus statement which suggests, when high-quality prostate imaging is available, an mpMRI for patients with a prior negative biopsy but with a persistent clinical suspicion for Pca [22]. In addition, even in those patients who will undergo a repeated biopsy, mpMRI could further improve the detection rate of PCa, permitting us to perform a targeted (fusion or cognitive) biopsy and increasing overall detection rates, as confirmed by Abdi et al. in a recent study involving 283 patients with a previous negative biopsy [23,24]. Similar results were reported by Hambrock et al., which showed a superior PCa detection rate of mpMRI targeted biopsy compared to systematic TRUS-guided biopsy in male with previous negative biopsy, reporting a detection rate of 59%, using targeted biopsy with a median of four cores on a total of 71 patients, with a clinical suspicion of PCa confirmed at mpMRI [25]. Additionally, Portalez et al. found that targeted cores reported an higher rate of PCa detection compared to systemic biopsy alone (36.3% versus 4.9%), while Costa et al. reported a higher yield of positive biopsy in patients with previous negative biopsy undergoing mpMRI compared to those undergoing systematic biopsy alone (92% versus 23) [26,27]. Interestingly, 77% of PCa were exclusively detected in zones highlighted at mpMRI [27]. Lastly, a systematic review and meta-analysis by Schoots et al. reported an improved overall PCa and csPCa detection rates of MRI-targeted biopsy compared to with systematic TRUS-guided biopsy in patients with previous negative biopsy [28]. More recently, different studies have considered the role of mpMRI in patients with a previous negative biopsy. Patel et al. evaluated, on a large sample size of 900 men (420 with a prior negative biopsy and 480 biopsy-naïve), the detection rate of mpMRI, reporting an overall lower risk of detection of any PCa and csPCa in the first group, with 27.9% versus 54.4% and 20% versus 38.3%, respectively, resulting in a 45.8% reduction of the number of unnecessary biopsies [29]. A previous study by Truong et al., involving 285 patients with a previous negative biopsy, similarly reported a decreased risk of PCa detection, with a false positive mpMRI occurring in up to 46.3% of patients [30]. In addition, Ryoo et al. reported in 497 patients with a previous negative biopsy, a csPCa in 3% of patients with a PIRADS 1 or 2, and 6.5% in PIRADS 3, while comparable results with biopsy-naïve patients were reported for PIRADS 4 and 5 [31]. According to the current literature, we reported a lower risk of any PCa and csPCa for patients who underwent a previous negative biopsy compared to biopsy-naïve patients. However, it has to be also noted that, although a slightly higher rate of false-positive was reported for PIRADS 4 and 5, the efficacy of mpMRI in diagnosing csPCa was comparable in both groups.

Performing mpMRI before a repeated biopsy harbors several advantages. The use of mpMRI to select patients for repeated biopsy, in addition to increasing the detection rate of any PCa and csPCa compared to TRUS biopsy alone, could reduce (up to 73%) the number of biopsies needed, lowering the costs per detection compared to TRUS [32]. Additionally, the possibility to highlight suspicious zones at the mpMRI represents another advantage in performing, successively, systematic, or targeted biopsy, permitting us to obtain cores from the prostate zone interested or signaled at the imaging.

We are conscious of the different limitations of our study. Firstly, the retrospective analysis could not eliminate the potential selection bias; secondly, a clear and widespread definition of csPCa has not yet been achieved; thirdly, the relatively small sample size of patients involved in the study; fourthly, the lack of a histopathological analysis performed on the specimen after radical prostatectomy. Nevertheless, our study could further enrich the literature on the topic, highlighting the necessity of implement novel protocols in the follow-up and management of patients with previous negative biopsy. Future studies are undoubtedly required to properly evaluate the potential improvement of the diagnostic accuracy of mpMRI also in this kind of patients. Additionally, an implementation of imaging, PSA values, and novel methodologies (for example liquid biopsy) should be considered in the overall clinical management of suspicious PCa. In this regard, the novel field of radiomics and machine learning applied to mpMRI could represent a further aid towards this objective [33,34,35,36].

## 5. Conclusions

mpMRI is equally able to detect significant PCa in biopsy-naïve males, as well as in males with prior negative biopsies. mpMRI permits safely ruling out clinically significant results and prostate cancer, potentially limiting the necessity of performing a further prostate biopsy.

## Figures and Tables

**Table 1:** Descriptive characteristics of total patients involved. PSA: prostate specific antigen; DRE: digital rectal exam; PIRADS: Prostate Imaging—Reporting and Data System; ISUP: International Society of Urological Pathology.

	Mean	Standard Deviation
**Age**	68.07	7.02
**PSA (ng/mL)**	9.02	7.12
**Number of cores**	15.76	1.26
	**Count**	**Percentage**
**Positive DRE**	172	44.3
**PIRADS score**		
2	56	14.4
3	124	31.9
4	137	35.2
5	72	18.5
**Side at mpMRI**		
Bilateral	47	12.1
Left	170	43.7
Right	160	41.1
None	12	3.1
**Gleason Score**		
Negative	186	47.8
PIN	7	1.8
3 + 3	53	13.6
3 + 4	22	5.7
4 + 3	8	2.1
4 + 4	91	23.4
4 + 5	7	1.8
5 + 4	11	2.8
5 + 5	4	1
**Side at Biopsy**		
Bilateral	57	14.7
Left	72	18.5
Right	74	19
None	186	47.8
**ISUP Grade**		
1	60	29.6
2	22	10.8
3	8	3.9
4	91	44.8
5	22	10.8
**Complications (Clavien-Dindo)**		
1	17	4.4
2	14	3.6
3	8	2.1

**Table 2:** Comparison between biopsy-naïve patients (Group A) and re-biopsy patients (Group B). * Statistically significant. PSA: prostate specific antigen; DRE: digital rectal exam; PIRADS: Prostate Imaging—Reporting and Data System; ISUP: International Society of Urological Pathology.

	Group A (Biopsy-Naïve) = 327	Group B (Re-Biopsy) = 62	*p* Value
	Mean ± Standard Deviation	Mean ± Standard Deviation	
**Age**	68.05 ± 7.10	68.18 ± 6.63	0.828
**PSA (ng/mL)**	8.91 ± 7.31	9.59 ± 5.96	0.624
**Number of cores**	15.69 ± 1.13	16.13 ± 1.76	0.661
	**Count (Percentage)**	**Count (Percentage)**	
**Positive DRE**	137 (79.6)	35 (20.4)	0.047 *
**PIRADS score**			
2	45 (13.8)	11 (17.7)	0.683
3	103 (31.5)	21 (33.9)	0.683
4	119(36.4)	18 (29)	0.683
5	60 (18.4)	12 (19.4)	0.683
**Side at mpMRI**			
Bilateral	37 (11.3)	10 (16.1)	0.467
Left	147 (45)	23 (37.1)	0.467
Right	132 (40.4)	28 (45.2)	0.467
None	11 (3.4)	1 (1.6)	0.467
**Gleason Score**			
Negative	155 (47.4)	31 (50)	0.635
PIN	5 (1.5)	2 (3.2)	0.635
3 + 3	45 (13.8)	8 (12.9)	0.635
3 + 4	19 (5.8)	3 (4.8)	0.635
4 + 3	5 (1.5)	3 (4.8)	0.635
4 + 4	78 (23.9)	13 (21)	0.635
4 + 5	7 (2.1)	0 (0)	0.635
5 + 4	9 (2.8)	2 (3.2)	0.635
5 + 5	4 (1.2)	0 (0)	0.635
**Side at Biopsy**			
Bilateral	49 (15)	8 (12.9)	0.472
Left	64 (19.6)	8 (12.9)	0.472
Right	59 (18)	15 (24.2)	0.472
None	155 (47.4)	31 (50)	0.472
**ISUP Grade**			
1	50 (29.1)	10 (32.3)	0.562
2	19 (11)	3 (9.7)	0.562
3	5 (2.9)	3 (9.7)	0.562
4	78 (45.3)	13 (41.9)	0.562
5	20 (11.6)	2 (6.5)	0.562
**Complications (Clavien-Dindo)**			
1	15 (44.1)	2 (40)	0.978
2	12 (35.3)	2 (40)	0.978
3	7 (20.6)	1 (20)	0.978

**Table 3:** Concordance between PIRADS groups and histopathologic results at biopsy among biopsy-naïve patients (Group A) (*p* < 0.0001) and re-biopsy patients (Group B) (*p* = 0.040) (percentages refer to PIRADS groups).

	Negative		PIN		GS 3 + 3		GS 3 + 4		GS 4 + 3		GS 4 + 4		GS 4 + 5		GS 5 + 4		GS 5 + 5	
	Group A	Group B	Group A	Group B	Group A	Group B	Group A	Group B	Group A	Group B	Group A	Group B	Group A	Group B	Group A	Group B	Group A	Group B
**PIRADS 2**	28 (62.2)	10 (90.9)	0 (0)	0 (0)	13 (28.9)	1 (9.1)	3 (6.7)	0 (0)	0 (0)	0 (0)	0 (0)	0 (0)	1 (2.2)	0 (0)	0 (0)	0 (0)	0 (0)	0 (0)
**PIRADS 3**	64 (62.1)	10 (47.6)	2 (1.9)	1 (4.8)	19 (18.4)	5 (23.8)	9 (8.7)	2 (9.5)	1 (1)	1 (4.8)	7 (6.8)	2 (9.5)	1 (1)	0 (0)	0 (0)	0 (0)	0 (0)	0 (0)
**PIRADS 4**	58 (48.7)	9 (50)	3 (2.5)	1 (5.6)	12 (10.1)	1 (5.6)	3 (2.5)	0 (0)	4 (3.4)	2 (11.2)	35 (29.4)	4 (22.2)	1 (0.8)	0 (0)	2 (1.7)	1 (5.6)	1 (0.8)	0 (0)
**PIRADS 5**	5 (8.3)	2 (16.7)	0 (0)	0 (0)	1 (1.7)	1 (8.3)	4 (6.7)	1 (8.3)	0 (0)	0 (0)	36 (60)	7 (58.3)	4 (6.7)	0 (0)	7 (11.7)	1 (8.3)	3 (5)	0 (0)

**Table 4:** Concordance between PIRADS groups and ISUP grade at biopsy among biopsy-naïve patients (Group A) (*p* < 0.0001) and re-biopsy patients (Group B) (*p* = 0.018) (percentages refer to PIRADS groups).

ISUP	0		1		2		3		4		5	
	Group A	Group B	Group A	Group B	Group A	Group B	Group A	Group B	Group A	Group B	Group A	Group B
**PIRADS 2**	28 (62.2)	10 (90.9)	13 (28.9)	1 (9.1)	3 (6.7)	0 (0)	0 (0)	0 (0)	0 (0)	0 (0)	1 (2.2)	0 (0)
**PIRADS 3**	64 (62.1)	10 (47.6)	21 (20.4)	6 (28.6)	9 (8.7)	2 (9.5)	1 (1)	1 (9.1)	7 (6.8)	2 (9.5)	1 (1)	0 (0)
**PIRADS 4**	58 (48.7)	9 (50)	15 (12.6)	2 (11.1)	3 (2.5)	0 (0)	4 (3.4)	2 (22.2)	35 (29.4)	4 (22.2)	4 (3.4)	1 (5.6)
**PIRADS 5**	5 (8.3)	2 (16.7)	1 (1.7)	1 (8.3)	4 (6.7)	1 (8.3)	0 (0)	0 (0)	36 (60)	7 (58.3)	14 (23.3)	1 (8.3)

## Data Availability

Data are available on request.
